# Association of ERCC1 C8092A and ERCC2 Lys751Gln Polymorphisms with the Risk of Glioma: A Meta-Analysis

**DOI:** 10.1371/journal.pone.0095966

**Published:** 2014-04-24

**Authors:** Yu Xin, Shuyu Hao, Jiapeng Lu, Qianyi Wang, Liwei Zhang

**Affiliations:** 1 Department of Neurosurgery, Beijing Tiantan Hospital, Capital Medical University, Dongcheng District, Beijing, People's Republic of China; 2 National Clinical Research Center of Cardiovascular Diseases, State Key Laboratory of Cardiovascular Disease, Fuwai Hospital, National Center for Cardiovascular Diseases, Chinese Academy of Medical Sciences and Peking Union Medical College, Beijing, People's Republic of China; 3 Faculty of Economics and Administration, University of Malaya, Kuala Lumpur, Malaysia; Sudbury Regional Hospital, Canada

## Abstract

**Objectives:**

To comprehensively evaluate the association of ERCC1 C8092A and ERCC2 Lys751Gln polymorphisms with the risk of glioma.

**Methods:**

Potential studies were searched and selected through the Pubmed/MEDLINE, EMBASE, the China National Knowledge Infrastructure (CNKI) platforms, WanFang and VIP database up to June 2013. Two investigators independently reviewed full text and included studies met inclusion criteria. Combined odds ratios (ORs) and 95% confidence intervals (95% CIs) were calculated in a fixed-effects model or a random-effects model according to results of heterogeneity test. All analyses were performed by Revman 5.2 and Stata 10.0 software.

**Results:**

A total of 10 studies were included in our meta-analysis, including 3,580 glioma patients and 4,728 controls. Overall, ERCC1 C8092A polymorphism was associated with the risk of glioma (AA vs. CC: OR = 1.29, 95%CI: 1.07–1.55, *P* = 0.01; recessive model: OR = 1.29; 95% CI: 1.07–1.55, *P* = 0.01). When stratified by ethnicity, significant association was only observed in the Chinese population (AA vs. CC: OR = 1.37, 95%CI: 1.03–1.81, *P* = 0.03; recessive model: OR = 1.34; 95% CI: 1.02–1.75, *P* = 0.04). For ERCC2 Lys751Gln polymorphism, no significant association was found between ERCC2 Lys751Gln polymorphism and the risk of glioma in different genetic models. A significant association of ERCC2 Lys751Gln polymorphism with the risk of glioma was identified in the Caucasian population under recessive model (OR = 0.87; 95% CI: 0.78–0.98, *P* = 0.02), but not in the Chinese population.

**Conclusion:**

This meta-analysis suggested that the AA genotype of ERCC1 C8092A polymorphism might increase the susceptibility of glioma in the Chinese population. And the TT genotype of ERCC2 Lys751Gln polymorphism may decrease the risk of glioma in the Caucasian population. But the small number of studies and moderate methodological quality require cautious interpretation of the study results.

## Introduction

Glioma is one of the most common brain tumors, accounting for approximately 80% of all brain tumors [1]. In a multi-center cross-sectional study in China, it is estimated that the age-standardized prevalence of primary brain tumor is 22.52 per 100,000 (95%CI: 13.22–31.82) for the entire population, and the overall prevalence in females (27.94 per 100,000) was higher than that in males (17.64 per 100,000). Of 272 newly diagnosed brain tumors, glioma accounted for 29.78% [2]. Although the prevalence of glioma is relatively lower than other cancers, survival rates of glioma patients is still poor, especially the 5 year survival rate of glioblastoma case, less than 3% [3], [4], [5].

Like other cancers, the development and progression of glioma are also determined by genetic and environmental factors. Currently, there are several confirmed environmental risk factors, including ionizing radiation, ultraviolet (UV) rays, diet and smoking. It is evidenced that the low exposure of ionizing radiation and ultraviolet (UV) rays would reduce the susceptibility of glioma [6], [7]. Ionizing radiation and UV rays can result in several kinds of DNA damage, such as oxidative DNA damage, and single- and double-strand breaks in DNA chains [8]. Consequently, DNA damages would result in glioma development and progression. However, major DNA repair pathways exist in the human body for preventing DNA damage and mutagenesis including base-excision repair (BER), nucleotide excision repair (NER), and homologous recombination repair (HRR) [9]. Due to the strong contributions to genomic integrity and fidelity that DNA repair genes encompass, many studies have focused on polymorphisms within them in relation to glioma susceptibility. Among these genes, excision repair cross-complementing group 1 (ERCC1) and ERCC2 gene are considered to be related with the susceptibility of glioma due to their roles as rate limiting enzymes in association to NER. The NER pathway shows effective and prominent repair on bulky DNA lesions and UV damage [10], [11].

Several single nucleotide polymorphisms (SNPs) have been identified in ERCC1 and ERCC2 genes. But ERCC1 C8092A and ERCC2 Lys751Gln polymorphisms have significant influences on gene expression and DNA repair functions. ERCC1 C8092A polymorphism, located in the 3′-untranslated region of the ERCC1 gene, has been shown to influence the level of ERCC1 mRNA expression and may affect mRNA stability for ERCC1 [12], [13]. And individuals with ERCC2 Lys/ Lys 751 genotype had poorer repair proficiency based on the number of chromatid aberrations (OR = 7.5, 95%CI = 1.01–87.7), suggesting this polymorphism is associated with an increased risk of suboptimal DNA repair [14], [15]. These two polymorphisms have been widely investigated in different ethnic populations. In a study on adult glioma cases and controls in a Caucasian population, it was presented that patients with oligoastrocytoma were significantly more likely to be homozygous in ERCC1 C8092A polymorphism than the controls (CC versus CA/AA: OR = 4.6, 95% CI: 1.6–13.2), however, no significant results were found in other histologic types of glioma [16]. Another study in the Caucasian population observed that ERCC1 C8092A polymorphism was associated with risk of glioma in recessive model (AA versus CC/CA: OR = 1.86, 95%CI: 1.01–3.46) [17], however, no significant findings were reported in other studies in the Caucasian or other ethnic populations [18], [19], [20]. In a Chinese population the variants of ERCC2 Lys751Gln polymorphism was significantly different between glioma cases and controls (χ^2^ = 6.015, *P* = 0.014) [21]. In addition, among Caucasians, glioma cases were significantly more likely than controls to be homozygous for ERCC2 Lys751Gln polymorphism (OR = 3.2, 95% CI: 1.1–9.3) [22]. However, other association studies did not show any positive results [17], [18], [23].

Evidently, numerous studies have been done to identify the association of ERCC1 C8092A and ERCC2 Lys751Gln polymorphisms with the risk of glioma in different ethnic populations. But the findings were not consistent. In this study, we performed a meta-analysis of the available studies in different ethnic populations to evaluate the effects of ERCC1 C8092A and ERCC2 Lys751Gln polymorphisms on the susceptibility to glioma.

## Materials and Methods

### Publication searching

We searched for relevant studies up to June 2013 in both English and Chinese through the Pubmed/MEDLINE, EMBASE, the China National Knowledge Infrastructure (CNKI) platforms, WanFang and VIP database with the following terms and their combinations: “ERCC1”, “ERCC2 or XPD” or “ERCC”, “glioma” and “polymorphism or variant”. We tried to identify potential relevant studies from the whole reference lists by orderly reviewing title, abstract and full text.

### Selection criteria

The inclusion criteria were as follows: a). Research focused on the association of ERCC1 C8092A (rs3212986) or ERCC2 Lys751Gln (rs13181) polymorphisms with the risk of glioma; b). Case-control studies; c). Genotype and allele data available. Studies were excluded for following reasons: a). unpublished papers, dissertations, conference articles, reviews and duplication of publications (select the study in a larger sample size); b). data unavailable for calculating genotype or allele frequencies; c). genotype distribution of control subjects violates the Hardy–Weinberg equilibrium (HWE).

### Data extraction

All the following information was separately extracted by two investigators, including: the first author, year of publication, country (ethnicity), allele frequencies and genotype distributions in glioma cases and controls, number of glioma cases and controls. Extracted data were compared and cross referenced. Inconsistencies were discussed and corrected together.

### Quality assessment

We evaluated the methodological quality of included studies using the Strengthening the Reporting of Genetic Association studies (STREGA) recommendations, extended the Strengthening the Reporting of Observational Studies in Epidemiology (STROBE) checklist [24]. The resulting STREGA checklist provides additions to 12 of the 22 items on the STROBE checklist. Two investigators independently assessed the quality of all included studies to determine whether they meet each item or not.

### Data analysis

All meta-analysis was performed by Review manager 5.2 software (The Cochrane Information Management System). The pooled odds ratios (OR) and 95% confidence interval (CI) were calculated for measuring the genetic association between ERCC1 or ERCC2 polymorphisms and the risk of glioma. The between-study heterogeneity and the *I* ^2^ statistic for estimation of inconsistency was analyzed using the heterogeneity Q statistic test. The following cut-off points indicated different degrees of heterogeneity: *I* ^2^ = 0–25%, no heterogeneity; *I* ^2^ = 25–50%, moderate heterogeneity; *I* ^2^ = 50–75%, large heterogeneity; *I* ^2^ = 75–100%, extreme heterogeneity [25]. If there was no significant between-study heterogeneity (*P* ≥ 0.10), the method of Mantel-Haenszel was used to calculate the pooled OR (95%CI) in a fixed effects model. Otherwise, the DerSimonian-Laird method was performed for evaluating the pooled OR (95%CI) in a random effects model. Begg's funnel plot and Egger's test were performed to assess publication bias among the literatures by Stata 10 software. Funnel plots were used to evaluate publication bias. *P*<0.05 was considered statistically significant.

## Results

### Characteristics of eligible publications

**Figure 1:**
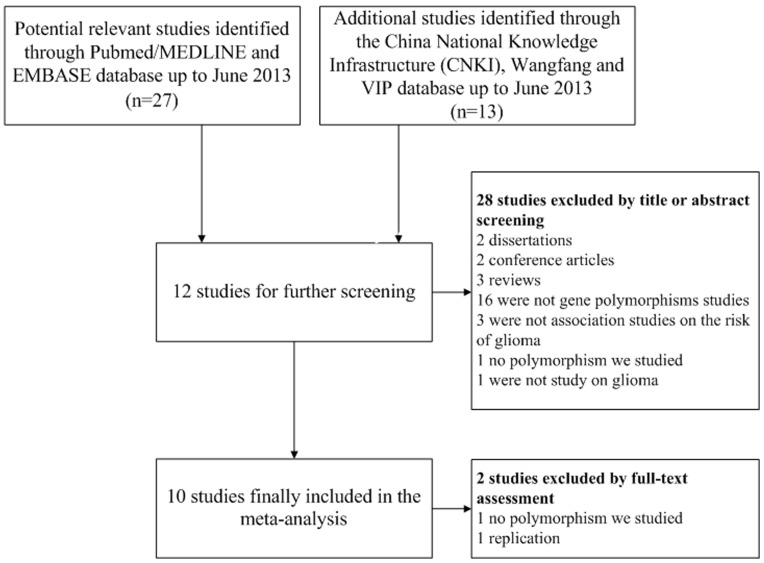
Flow chart of study selection.

The initial search identified a total of 40 studies for ERCC1 C8092A and ERCC2 Lys751Gln, of which 10 studies met the selection criteria [16], [17], [18], [19], [20], [21], [22], [23], [26], [27]. Among the 30 excluded articles, two were dissertations, two were conference articles, three were reviews, 16 were not gene polymorphism studies, three were not association studies on the risk of glioma, two were not the polymorphism we studied, one was not a study on glioma and one was a replication (Figure 1).

Among the 10 selected studies, seven studies reported ERCC1 C8092A, including 2,936 glioma cases and 4,017 controls (Table 1) [16], [17], [18], [19], [20], [22], [23]. There were three studies in the Chinese population [19], [20], [23] and four studies in the Caucasian population [16], [17], [18], [22]. In the seven studies, the frequency of ERCC1 8092A allele was 27.08% for glioma cases and 25.45% for controls.

**Table 1:** Characteristics of seven eligible studies for ERCC1 C8092A and ERCC2 Lys751Gln polymorphisms.

First Author	Year	Country	Ethnicity	Age (Mean±SD)		Gender (%)	Glioma cases				Controls				*P* for HWE	Quality score
				Case	Control	Case/Control	CC	CA	AA	Total	CC	CA	AA	Total		
ERCC1 C8092A																
Chen [16]	2000	USA	Caucasian	49.6±1.3	53.2±1.2	61.0/54.0	73	43	6	122	81	70	8	159	0.145	15
Chen [23]	2012	China	Chinese	50.4±7.9	49.6±8.5	61.5/62.0	202	141	50	393	221	154	35	410	0.273	17
Liu [17]	2009	USA	Caucasian	NA	NA	58.6/43.6	208	130	31	369	219	126	17	362	0.836	17
McKean-Cowdin [18]	2009	USA	Caucasian	56.3±12.6	53.6±15.3	61.0/51.1	557	361	59	977	1087	728	105	1920	0.237	18
Pan [19]	2013	China	Chinese	50.9±9.6	51.2±9.1	58.0/58.0	229	169	45	443	241	162	41	444	0.075	17
Wrensch [22]	2005	USA	Caucasian	51±0.67	56±0.67	41.0/46.0	206	144	25	375	237	184	23	444	0.093	18
Zhang [20]	2012	China	Chinese	47.6±7.5	46.8±6.2	62.3/62.2	123	98	36	257	144	105	29	278	0.139	19
ERCC2 Lys751Gln																
Caggana [26]	2001	USA	Caucasian	48.1±1.2	53.3±1.2	61.0/54.0	23	63	62	148	23	76	49	148	0.467	16
Chen [23]	2012	China	Chinese	50.4±7.9	49.6±8.5	61.5/62.0	139	198	56	393	175	186	49	410	0.969	17
Liu [17]	2009	USA	Caucasian	NA	NA	58.6/43.6	56	172	139	367	45	156	161	362	0.452	17
McKean-Cowdin [18]	2009	USA	Caucasian	56.3±12.6	53.6±15.3	61.0/51.1	143	480	376	999	256	891	823	1970	0.542	18
Rajaraman [27]	2010	USA	Caucasian	51.2	49.2	54.7/46.1	52	171	128	351	66	215	200	481	0.499	17
Wrensch [22]	2005	USA	Caucasian	51±0.67	56±0.67	41.0/46.0	57	169	139	365	55	213	164	432	0.269	18
Yang [21]	2005	China	Chinese	NA	NA	NA	0	32	103	135	0	3	41	44	0.815	12

MAF: Minor Allele Frequency; HWE: Hardy-Weinberg equilibrium; NA: Not Available.

Likewise, seven studies reported ERCC2 Lys751Gln, including 2,758 glioma cases and 3,847 controls (Table 1) [17], [18], [21], [22], [23], [26], [27]. There were two studies in the Chinese population [21], [23], while five studies in the Caucasian population [17], [18], [22], [26], [27]. The overall frequency of ERCC2 751Gln allele was 59.66% for glioma cases and 61.27% for controls.

The genetic distributions of ERCC1 C8092A and ERCC2 Lys751Gln polymorphisms in the all studies followed the law of HWE (*P* > 0.05). The methodological quality scores of all the included studies were from 12 to 19, indicating that all the included studies have moderate or high quality.

### Main results of meta-analysis

#### ERCC1 C8092A

**Figure 2:**
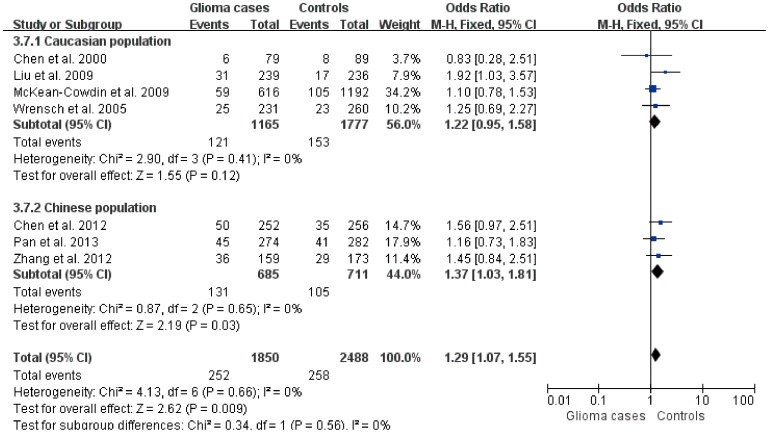
Forest plot of the subgroup analysis on the association of ERCC1 C8092A polymorphism with glioma (AA vs CC).

**Figure 3:**
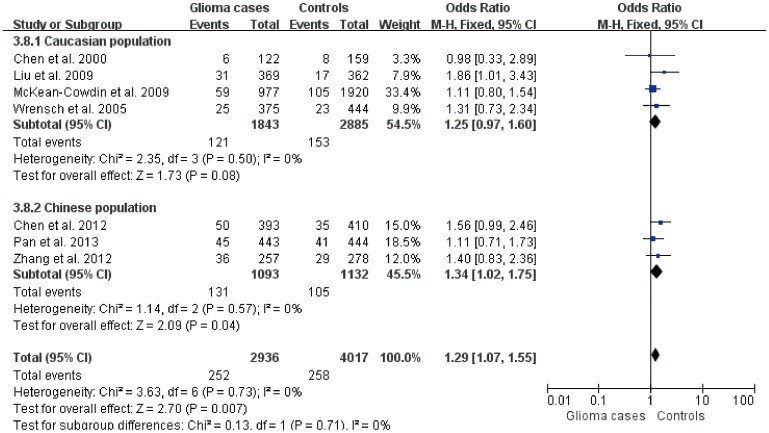
Forest plot of the subgroup analysis on the association of ERCC1 C8092A polymorphism with glioma (AA vs C-carriers).

The between-study heterogeneity was not significant in all the comparisons (*P* > 0.1). In addition, no between-study heterogeneity was identified using *I* ^2^ statistic (Table 2). Therefore, we used the fixed effects model to calculate the pooled OR (95%CI). A significant association was identified comparing AA with CC (OR = 1.29, 95%CI: 1.07–1.55, *P* = 0.01). Significant results were also observed in recessive model (OR = 1.29; 95% CI: 1.07–1.55, *P* = 0.01). There was no significant association in other genetic models. Subgroup analysis showed that ERCC1 8092AA was significantly associated with the risk of glioma compared with ERCC1 8092CC in Chinese population (Z = 2.19, *P* = 0.03), but was not found in Caucasian population (Z = 1.55, *P* = 0.12) (Figure 2). Similar results were obtained in recessive model (Figure 3).

**Table 2:** Meta-analysis of the association of ERCC1 C8092A and ERCC2 Lys751Gln polymorphism with the risk of glioma.

	Pooled OR	95% CI	Z	*P*	*I* ^2^	Heterogeneity chi-square	*P*'
ERCC1 C8092A							
A vs C	1.06	0.95–1.19	1.11	0.27	0	3.54	0.74
CA vs CC	0.99	0.89–1.09	0.28	0.78	0	3.83	0.70
AA vs CC	1.29	1.07–1.55	2.62	0.01*	0	4.13	0.66
Dominant (A-carriers vs CC)	1.03	0.93–1.13	0.58	0.56	0	5.31	0.50
Recessive (AA vs C-carriers)	1.29	1.07–1.55	2.70	0.01*	0	3.63	0.73
ERCC2 Lys751Gln							
T vs G	0.93	0.84–1.03	1.45	0.15	30	8.54	0.20
GT vs GG	1.00	0.87–1.16	0.04	0.97	15	5.89	0.32
TT vs GG	0.88	0.75–1.03	1.59	0.11	33	7.46	0.19
Dominant (T-carriers vs GG)	0.97	0.84–1.11	0.50	0.62	38	8.01	0.16
Recessive (TT vs G-carriers)	0.91	0.76–1.10	0.97	0.33	56	13.63	0.03^#^

OR: odds ratio; CI: confidence interval; *P*' was for heterogeneity test. * *P*<0.05, it was considered statistically significant.

#### ERCC2 Lys751Gln

**Figure 4:**
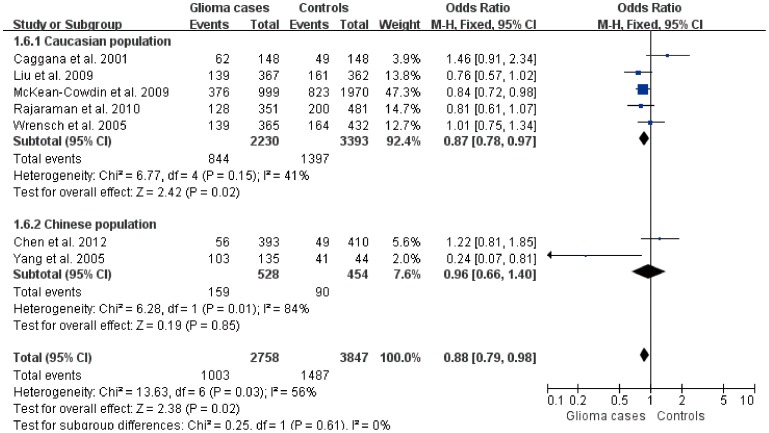
Forest plot of the subgroup analysis on the association of ERCC2 Lys751Gln polymorphism with glioma (TT vs G-carriers).

For the recessive model, the between-study heterogeneity was significant (*P* = 0.03), and large between-study heterogeneity was identified by *I* ^2^ statistic (Table 2). Therefore, the random effects model was used to calculate the pooled OR (95%CI) for the recessive model. For other genetic models, the fixed effects model was performed for calculating pooled OR (95%CI). No significant association was found between ERCC2 Lys751Gln polymorphism and the risk of glioma in different genetic models. However, subgroup analysis showed that a significant association was identified comparing TT with G-carriers in the Caucasian population (Z = 2.42, *P* = 0.02), but this was not found in the Chinese population (Z = 0.19, *P* = 0.85) (Figure 4).

Next, stratified analyses for different types of glioma including glioblastoma multiforme and nonglioblastoma multiforme were performed in ERCC2 Lys751Gln polymorphism. There was no significant association in both glioblastoma multiforme and nonglioblastoma multiforme groups (Table 3).

**Table 3:** Stratified analysis of the association of ERCC2 Lys751Gln polymorphism with the risk of different type of glioma.

	Pooled OR	95% CI	Z	*P*	*I* ^2^ (%)	Heterogeneity chi-square	*P*'
**Glioblastoma multiforme**							
T vs G	1.07	0.80–1.44	0.47	0.64	0	0.52	0.47
GT vs GG	0.83	0.60–1.14	1.17	0.24	0	0.02	0.89
TT vs GG	1.24	0.81–1.92	0.98	0.33	0	0.17	0.68
Dominant (T-carriers vs GG)	0.92	0.68–1.24	0.57	0.57	0	0.22	0.64
Recessive (TT vs G-carriers)	1.42	0.98–2.04	1.86	0.06	0	0.30	0.59
**Nonglioblastoma multiforme**							
T vs G	1.03	0.77–1.36	0.18	0.86	0	0.10	0.76
GT vs GG	0.87	0.64–1.18	0.89	0.37	0	0.05	0.82
TT vs GG	1.12	0.74–1.72	0.55	0.59	0	0.00	0.97
Dominant (T-carriers vs GG)	0.93	0.69–1.24	0.49	0.62	0	0.00	0.99
Recessive (TT vs G-carriers)	1.26	0.88–1.79	1.26	0.21	0	0.10	0.75

OR: odds ratio; CI: confidence interval; *P*' was for heterogeneity test. ^#^ *P*'<0.1, it was considered statistically significant.

### Sensitivity analysis

To further confirm the combined results, a sensitivity analysis was conducted by changing the fixed or random effects model. It was shown that the combined results between fixed effects model and random effects model for ERCC1 C8092A polymorphism were no different. However, a significant change was identified in recessive model for ERCC2 Lys751Gln polymorphism. The combined OR (95%CI) was 0.88 (0.79–0.98) estimated by fixed effects model (Z = 2.38, *P* = 0.02). We then estimated the influence of individual studies on the combined results by omitting one study at a time. No significant differences were found in different genetic models for ERCC1 C8092A polymorphism. When deleting a study in the Chinese population [23], the ERCC2 Lys751Gln polymorphism showed significant effects on the risk of glioma, but the significance was much less (Z = 2.10, *P* = 0.04, OR = 0.89, 95%CI: 0.79–0.99). Therefore, our results were generally and statistically reliable.

### Publication bias

**Figure 5:**
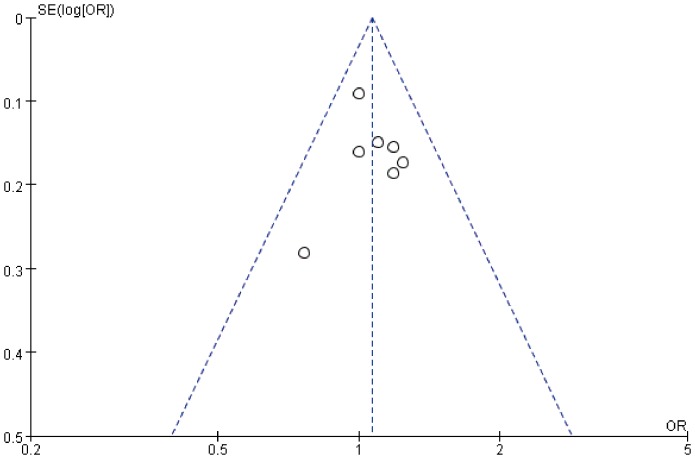
Funnel plot for ERCC1 C8092A polymorphism (C vs A).

**Figure 6:**
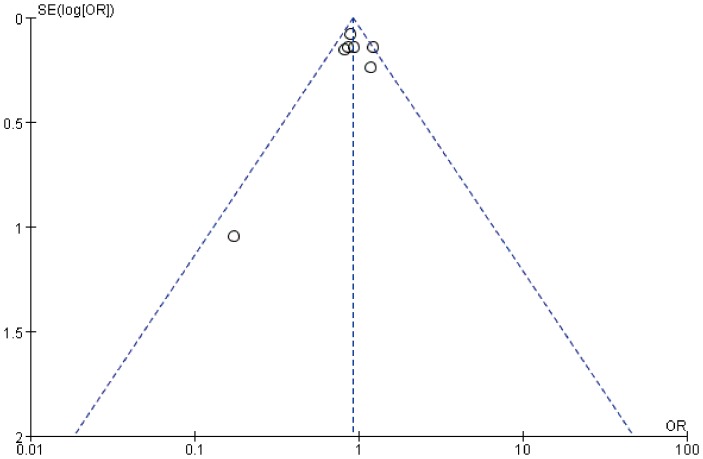
Funnel plot for ERCC2 Lys751Gln polymorphism (G vs T).

In this study, Begg's test and Egger's test did not show any evidence of publication bias for both ERCC1 C8092A (Begg's test: z = 0.00, *P*  = 1.00; Egger's test: t = 1.55, *P* = 0.18) and ERCC2 Lys751Gln polymorphisms (Begg's test: z = 0.30, *P* = 0.76; Egger's test: t = 1.03, *P* = 0.35). In the funnel plot (Figure 5 and 6), studies of ERCC1 C8092A and ERCC2 Lys751Gln polymorphisms showed symmetric distribution. All studies were nearly located in the region of 95% CI, suggesting that publication bias was not significant in this meta-analysis.

## Discussion

ERCC1 and ERCC2 protein, key elements in transcription-coupled NER pathway, are absolutely critical for efficient DNA repair capacity. These two proteins are essential for the maintenance of genetic stability, thus, deficits in ERCC1 and ERCC2 proteins are associated with cancer susceptibility [28]. Studies have found that ERCC1 and ERCC2 polymorphisms are associated with high levels of DNA adducts or lower DNA repair capacity [14], [29], [30], [31]. Therefore, an increasing amount of studies attempt to identify the association of ERCC1 and ERCC2 polymorphisms on the susceptibility of glioma.

To comprehensively estimate the associations of ERCC1 C8092A and ERCC2 Lys751Gln polymorphisms with the risk of glioma in different ethnic populations, meta-analyses in 10 studies including 3,580 glioma patients and 4,728 controls was carried out. To our knowledge, this is to date the largest meta-analysis conducted for ERCC1 C8092A and ERCC2 Lys751Gln polymorphisms in glioma. The data from meta-analysis showed a significant increase in frequency of ERCC1 8092AA genotype in glioma patients than in controls, indicating that ERCC1 8092AA genotype contribute to increases the risk of glioma with combined OR of 1.29 (1.07–1.55). When the analysis was stratified by ethnicity, a significant association was identified in the Chinese population with combined OR of 1.37 (1.03–1.81). However, the significant association disappeared for Caucasians. Similar results were identified in the recessive model of ERCC1 C8092A polymorphism. Remarkably, none of the original researches in Chinese populations reported the significant association between ERCC1 C8092A polymorphism and the risk of glioma. Therefore, the positive results of this meta-analysis might have been caused by small sample size. However, ERCC1 C8092A polymorphism influence the expression of ERCC1 and the DNA repair function of NER pathway may be weakened, a possible explanation of our findings from the point of the pathogenesis [13].

For ERCC2 Lys751Gln polymorphism, no significant association between ERCC2 Lys751Gln polymorphism and the risk of glioma was observed. However, TT genotype in ERCC2 Lys751Gln polymorphism may significantly decrease the risk of glioma in the Caucasian population with combined OR of 0.87 (0.78–0.97), but not in the Chinese population. Although 2230 glioma patients and 3393 controls were included in the subgroup analysis in the Caucasian population, only one study with larger sample size presented significant results (OR = 0.84, 95%CI: 0.72–0.98), which may influence the combined results [18]. When this study was deleted from the subgroup analysis, significant results in the Caucasian population disappeared. Therefore, the finding between ERCC2 Lys751Gln polymorphism and the risk of glioma in Caucasian population should be addressed with caution. Furthermore, a stratified analysis for different types of glioma in ERCC2 Lys751Gln polymorphism was performed, and no significant association was identified in either groups. However, only two studies provided the available data for glioma classification. In addition, glioma patients were only separated into two groups, including glioblastoma multiforme and nonglioblastoma multiforme. Therefore, we cannot explicitly estimate the effects of ERCC2 Lys751Gln polymorphism on the risk of different kinds of glioma in our study.

In this study, the heterogeneity Q statistic test and the *I* ^2^ statistic were used to test the between-study heterogeneity. For all studies, the significant heterogeneity existed only under recessive model in ERCC2 Lys751Gln polymorphism. When the studies were stratified according to ethnicity, significant heterogeneity still existed in the Chinese population, but no statistically significant heterogeneity was observed in the Caucasian population. This suggests that significant heterogeneity might be attributed to differing ERCC2 Lys751Gln polymorphism by ethnicity.

Sensitivity analysis was performed by changing the fixed or random effects model and omitting one study each time to observe the change of effects. For ERCC1 C8092A polymorphism, no significant change was observed. However, the effect of ERCC2 Lys751Gln polymorphism on the risk of glioma changed significantly, though the combined OR was very close to 1. We then looked for publication bias using Begg's test, Egger's test and funnel plot. No significant publication bias existed in this meta-analysis. This indicated that the results in this meta-analysis were generally reliable.

Although publication bias was not identified in this meta-analysis, there are some potential limitations. First, only ten eligible studies were included in this meta-analysis. Therefore, in the subgroup analyses by ethnicity, the number of cases and controls was relatively small, which may lead to low statistical power in identifying the association. Furthermore, different pathological types of glioma were not considered, which may be the source of between-study heterogeneity. Unfortunately, only two studies in Caucasian populations provide genetic data of ERCC2 Lys751Gln polymorphism for glioblastoma multiforme and nonglioblastoma multiforme, respectively. Lastly, our meta-analysis was largely performed by unadjusted estimates, because of the limitations in selected studies that presented adjusted estimates. Although adjusted estimates were shown, the estimates were not adjusted by the same confounders. It was also difficult to present the combined estimates by adjusted potential confounders.

In conclusion, our meta-analysis suggested that ERCC1 8092AA genotype was associated with the higher susceptibility of glioma in the Chinese population. However, TT genotype of ERCC2 Lys751Gln polymorphism might decrease the risk of glioma in Caucasian population. Due to the small number of studies and moderate methodological quality, the results require cautious interpretation. Studies with larger sample size and more specified information in pathological types of glioma are needed to confirm our results for both Chinese and Caucasian populations.

## Supporting Information

Checklist S1 PRISMA checklist.
(DOC)
