# Supplementary material for: Association of ERCC1 C8092A and ERCC2 Lys751Gln Polymorphisms with the Risk of Glioma: A Meta-Analysis
Source: PLoS One. 2014 Apr 24;9(4):e95966. doi: 10.1371/journal.pone.0095966 (PMC3999106; doi:10.1371/journal.pone.0095966)
Supplement: Checklist S1 — PRISMA checklist. (DOC) [file pone.0095966.s001.doc]

| **Section/topic** | **#** | **Checklist item** | Reported on page # |
| --- | --- | --- | --- |
| **TITLE** | | |  |
| Title | 1 | Association of ERCC1 C8092A and ERCC2 Lys751Gln polymorphisms with the risk of glioma: A meta-analysis | Title |
| **ABSTRACT** | | |  |
| Structured summary | 2 | **Objectives:** To comprehensively evaluate the association of ERCC1 C8092A and ERCC2 Lys751Gln polymorphisms with the risk of glioma.  **Methods:** Potential studies were searched and selected through the Pubmed/MEDLINE, EMBASE, the China National Knowledge Infrastructure (CNKI) platforms, WanFang and VIP database up to June 2013. Two investigators independently reviewed full text and included studies met inclusion criteria. Combined odds ratios (ORs) and 95% confidence intervals (95% CIs) were calculated in a fixed-effects model or a random-effects model according to results of heterogeneity test. All analyses were performed by Revman 5.2 and Stata 10.0 software.  **Results:** A total of 10 studies were included in our meta-analysis, including 3,580 glioma patients and 4,728 controls. Overall, ERCC1 C8092A polymorphism was associated with the risk of glioma (AA vs. CC: OR = 1.29, 95%CI: 1.07-1.55, *P* = 0.01; recessive model: OR = 1.29; 95% CI: 1.07-1.55, *P* = 0.01). When stratified by ethnicity, significant association was only observed in the Chinese population (AA vs. CC: OR = 1.37, 95%CI: 1.03-1.81, *P* = 0.03; recessive model: OR = 1.34; 95% CI: 1.02-1.75, *P* = 0.04). For ERCC2 Lys751Gln polymorphism, no significant association was found between ERCC2 Lys751Gln polymorphism and the risk of glioma in different genetic models. A significant association of ERCC2 Lys751Gln polymorphism with the risk of glioma was identified in the Caucasian population under recessive model (OR = 0.87; 95% CI: 0.78-0.98, *P* = 0.02), but not in the Chinese population.  **Conclusion:** This meta-analysis suggested that the AA genotype of ERCC1 C8092A polymorphism might increase the susceptibility of glioma in the Chinese population. And the TT genotype of ERCC2 Lys751Gln polymorphism may decrease the risk of glioma in the Caucasian population. But the small number of studies and moderate methodological quality require cautious interpretation of the study results. | Abstract |
| **INTRODUCTION** | | |  |
| Rationale | 3 | Glioma is one of the most common brain tumors, accounting for approximately 80% of all brain tumors. The development and progression of glioma are also determined by genetic and environmental factors. Currently, there are several confirmed environmental risk factors, including ionizing radiation, ultraviolet (UV) rays, diet, smoking, and others. Excision repair cross-complementing group 1 (ERCC1) and ERCC2 gene are considered to be related with the susceptibility of glioma because their encoding proteins act as the rate-limiting enzymes in the process of NER. The NER pathway shows effective and prominent repair on bulky DNA lesions and UV damage | Introduction |
| Objectives | 4 | In this study, we performed a meta-analysis of the available studies in different ethnic populations to evaluate the effects of ERCC1 C8092A and ERCC2 Lys751Gln polymorphisms on the susceptibility to glioma | Introduction |
| **METHODS** | | |  |
| Protocol and registration | 5 | Pubmed/MEDLINE, EMBASE, the China National Knowledge Infrastructure (CNKI) platforms, WanFang and VIP database | Materials and Methods, Publication Searching |
| Eligibility criteria | 6 | The inclusion criteria were as follows: a). Research focused on the association of ERCC1 C8092A (rs3212986) or ERCC2 Lys751Gln (rs13181) polymorphisms with the risk of glioma; b). Case-control studies; c). Genotype and allele data available. Studies were excluded for following reasons: a). dissertations, conference articles, reviews and duplication of publications; b). data unavailable for calculating genotype or allele frequencies; c). genotype distribution of control subjects violates the Hardy–Weinberg equilibrium (HWE). | Materials and Methods, Selection criteria |
| Information sources | 7 | We searched for relevant studies up to June 2013 in both English and Chinese through the Pubmed/MEDLINE, EMBASE, the China National Knowledge Infrastructure (CNKI) platforms, WanFang and VIP database. | Materials and Methods, Publication Searching |
| Search | 8 | We searched for relevant studies up to June 2013 in both English and Chinese through the Pubmed/MEDLINE, EMBASE, the China National Knowledge Infrastructure (CNKI) platforms, WanFang and VIP database with the following terms and their combinations: “ERCC1”, “ERCC2 or XPD” or “ERCC”, “glioma” and “polymorphism or variant”. | Materials and Methods, Publication Searching |
| Study selection | 9 | We try to identify potential relevant studies in the whole reference lists by orderly reviewing title, abstract and full text. | Materials and Methods, Publication Searching |
| Data collection process | 10 | The initial search identified a total of 40 studies for ERCC1 C8092A and ERCC2 Lys751Gln, of which 10 studies met the selection criteria. Among the 30 excluded articles, two were dissertations, two were conference articles, three were reviews, 16 were not gene polymorphism studies, three were not association studies on the risk of glioma, two were not the polymorphism we studied, one was not a study on glioma and one was a replication (Figure 1). | Results, Characteristics of eligible publications |
| Data items | 11 | All the following information was separately extracted by two investigators, including: the first author, year of publication, country (ethnicity), allele frequencies and genotype distributions in glioma cases and controls, number of glioma cases and controls. Extracted data were compared and cross referenced. Inconsistencies were discussed and corrected together | Materials and Methods, Data extraction |
| Risk of bias in individual studies | 12 | None |  |
| Summary measures | 13 | All meta-analysis was performed by Review manager 5.2 software (The Cochrane Information Management System). The pooled odds ratios (OR) and 95% confidence interval (CI) were calculated for measuring the genetic association between ERCC1 or ERCC2 polymorphisms and the risk of glioma. *P* < 0.05 was considered statistically significant. | Materials and Methods, Data analysis |
| Synthesis of results | 14 | The between-study heterogeneity and the *I*^2^ statistic for estimation of inconsistency was analyzed using the heterogeneity Q statistic test. The following cut-off points indicated different degrees of heterogeneity: *I*^2^ = 0–25%, no heterogeneity; *I*^2^ = 25–50%, moderate heterogeneity; *I*^2^ = 50–75%, large heterogeneity; *I*^2^ = 75–100%, extreme heterogeneity. If there was significant between-study heterogeneity (*P* ≥ 0.10), the method of Mantel-Haenszel was used to calculate the pooled OR (95%CI) in a fixed effects model. Otherwise, the DerSimonian-Laird method was performed for evaluating the pooled OR (95%CI) in a random effects model. | Results, Main results of meta-analysis, ERCC1 C8092A |

Page 1 of 2

| **Section/topic** | **#** | **Checklist item** | Reported on page # |
| --- | --- | --- | --- |
| Risk of bias across studies | 15 | Begg’s funnel plot and Egger’s test were performed to assess the publication bias among the literatures by Stata 10 software. Funnel plots were used to evaluate the publication bias. | Materials and Methods, Data analysis |
| Additional analyses | 16 | To further confirm the combined results, a sensitivity analysis was conducted by changing the fixed or random effects model. We then estimated the influence of individual studies on the combined results by omitting one study at a time. | Materials and Methods, Sensitivity analysis |
| **RESULTS** | | |  |
| Study selection | 17 | The initial search identified a total of 40 studies for ERCC1 C8092A and ERCC2 Lys751Gln, of which 10 studies met the selection criteria. Among the 30 excluded articles, two were dissertations, two were conference articles, three were reviews, 16 were not gene polymorphism studies, three were not association studies on the risk of glioma, two were not the polymorphism we studied, one was not a study on glioma and one was a replication. The study selection process showed in Figure 1. | Results, Characteristics of eligible publications |
| Study characteristics | 18 | Among the 10 selected studies, seven studies reported ERCC1 C8092A, including 2,936 glioma cases and 4,017 controls (Table 1). There were three studies in the Chinese population and four studies in the Caucasian population. In the seven studies, the frequency of ERCC1 8092A was 27.08% for glioma cases and 25.45% for controls.  Likewise, seven studies reported ERCC2 Lys751Gln, including 2,758 glioma cases and 3,847 controls (Table 2). There were two studies in the Chinese population, while five studies in the Caucasian population. The overall frequency of ERCC2 751Gln was 59.66% for glioma cases and 61.27% for controls. | Results, Characteristics of eligible publications |
| Risk of bias within studies | 19 | None |  |
| Results of individual studies | 20 | We estimated the influence of individual studies on the combined results by omitting one study at a time. No significant differences were found in different genetic models for ERCC1 C8092A polymorphism. When deleting a study in the Chinese population[22], the ERCC2 Lys751Gln polymorphism showed significant effects on the risk of glioma, but the significance was much less (Z = 2.10, P = 0.04, OR = 0.89, 95%CI: 0.79-0.99). | Results, Sensitivity analysis |
| Synthesis of results | 21 | **ERCC1 C8092A**  The between-study heterogeneity was not significant in all the comparisons (*P* > 0.1). In addition, no between-study heterogeneity was identified using *I*^2^ statistic (Table 3). Therefore, we used the fixed effects model to calculate the pooled OR (95%CI). A significant association was identified comparing AA with CC (OR = 1.29, 95%CI: 1.07-1.55, *P* = 0.01). Significant results were also observed in recessive model (OR = 1.29; 95% CI: 1.07-1.55, *P* = 0.01). There was no significant association in other genetic models. Subgroup analysis showed that ERCC1 8092AA was significantly associated with the risk of glioma compared with ERCC1 8092CC in Chinese population (Z = 2.19, *P* = 0.03), but was not found in Caucasian population (Z = 1.55, *P* = 0.12) (Figure 2). Similar results were obtained in recessive model (Figure 3).  **ERCC2 Lys751Gln**  For recessive model, the between-study heterogeneity was significant (*P* = 0.03), and large between-study heterogeneity was identified by *I*^2^ statistic (Table 4). Therefore, the random effects model was used to calculate the pooled OR (95%CI) for recessive model. For other genetic models, the fixed effects model was performed for calculating pooled OR (95%CI). No significant association was found between ERCC2 Lys751Gln polymorphism and the risk of glioma in different genetic models. However, subgroup analysis showed that a significant association was identified comparing TT with G-carriers in the Caucasian population (Z = 2.42, *P* = 0.02), but this was not found in the Chinese population (Z = 0.19, *P* = 0.85) (Figure 4). | Results, Main results of meta-analysis |
| Risk of bias across studies | 22 | In this study, Begg’s test and Egger’s test did not show any evidence of publication bias for both ERCC1 C8092A (Begg’s test: z = 0.00, *P* =1.00; Egger’s test: t = 1.55, *P* = 0.18) and ERCC2 Lys751Gln (Begg’s test: z = 0.30, *P* = 0.76; Egger’s test: t = 1.03, *P* = 0.35) polymorphisms. In the funnel plot (Figure 5 and 6), studies of ERCC1 C8092A and ERCC2 Lys751Gln polymorphisms showed symmetric distribution. All studies were nearly located in the region of 95% CI, suggesting that publication bias was not significant in this meta-analysis. | Results, Publication bias |
| Additional analysis | 23 | Stratified analyses for different types of glioma including glioblastoma multiforme and nonglioblastoma multiforme were performed in ERCC2 Lys751Gln polymorphism. There was no significant association in both glioblastoma multiforme and nonglioblastoma multiforme groups (Table 5). | Results, Main results of meta-analysis, ERCC2 Lys751Gln |
| **DISCUSSION** | | |  |
| Summary of evidence | 24 | The data from meta-analysis showed a significant increase in frequency of ERCC1 8092AA genotype in glioma patients than in controls, indicating that ERCC1 8092AA genotype increases the risk of glioma with combined OR of 1.29 (1.07-1.55). When the analysis was stratified by ethnicity, a significant association was identified in the Chinese population with combined OR of 1.37 (1.03-1.81). However, the significant association disappeared for Caucasians. Similar results were identified in the recessive model of ERCC1 C8092A polymorphism. | Discussion |
| Limitations | 25 | First, only ten eligible studies were included in this meta-analysis. Therefore, in the subgroup analyses by ethnicity, the number of cases and controls was relatively small, which may lead to low statistical power in identifying the association. Furthermore, different pathological types of glioma were not considered, which may be the source of between-study heterogeneity. Unfortunately, only two studies in Caucasian populations provide genetic data of ERCC2 Lys751Gln polymorphism for glioblastoma multiforme and nonglioblastoma multiforme, respectively. Lastly, our meta-analysis was largely performed by unadjusted estimates, because of the limitations in selected studies that presented adjusted estimates. Although adjusted estimates were shown, the estimates were not adjusted by the same confounders. It was also difficult to present the combined estimates by adjusted potential confounders. | Discussion |
| Conclusions | 26 | In conclusion, our meta-analysis strongly suggested that ERCC1 8092AA genotype was associated with the higher susceptibility of glioma in the Chinese population. However, TT genotype of ERCC2 Lys751Gln polymorphism might decrease the risk of glioma in Caucasian population. Studies with larger sample size and more specified information in pathological types of glioma are needed to confirm our results for both Chinese and Caucasian populations. | Discussion |
| **FUNDING** | | |  |
| Funding | 27 | None |  |

*From:*  Moher D, Liberati A, Tetzlaff J, Altman DG, The PRISMA Group (2009). Preferred Reporting Items for Systematic Reviews and Meta-Analyses: The PRISMA Statement. PLoS Med 6(6): e1000097. doi:10.1371/journal.pmed1000097

For more information, visit: **www.prisma-statement.org**.

Page 2 of 2
